# *In vitro* evaluation of sodium butyrate on the growth of three *Salmonella* serovars derived from pigs at a mild acidic pH value

**DOI:** 10.3389/fvets.2022.937671

**Published:** 2022-07-26

**Authors:** Isabell Hollmann, Jan Berend Lingens, Bussarakam Chuppava, Volker Wilke, Amr Abd El-Wahab, Juhle Buch, Julia Hankel, Marwa F. E. Ahmed, Christian Visscher

**Affiliations:** ^1^Institute for Animal Nutrition, University of Veterinary Medicine Hannover, Foundation, Bischofsholer Damm 15, Hannover, Germany; ^2^Department of Nutrition and Nutritional Deficiency Diseases, Faculty of Veterinary Medicine, Mansoura University, Mansoura, Egypt; ^3^AniCon Labor GmbH, Höltinghausen, Germany; ^4^Hygiene and Zoonoses Department, Faculty of Veterinary Medicine, Mansoura University, Mansoura, Egypt

**Keywords:** *Salmonella*, pigs, butyrate, zoonotic diseases, one health, emerging infectious diseases

## Abstract

Foodborne zoonotic diseases can be transferred into the food chain at the stage of livestock farming. As an emerging public health challenge, practicable reduction measures in porcine health management for *Salmonella* are constantly being investigated. This *in vitro* study aimed to determine the influence of six different sodium butyrate (SB) concentrations (0, 5, 10, 20, 40, and 80 mM) on the growth of three different *Salmonella enterica* serovars at a constant pH value of 6.0, corresponding to conditions in the pig's hindgut. *S*. Derby and *S*. Typhimurium, isolated from a pig farm, and *S*. Typhimurium DSM 19587, which served as control, were used. Broth microdilution assay was applied to record *Salmonella* growth in the presence of different SB-concentrations over six different incubation periods (0, 1, 2, 4, 6, and 24 h). Results were quantified in the log colony-forming units (log_10_ CFU/mL). For 1 h incubation, the addition of SB showed no significant differences in the range of initial *Salmonella* dose of about 5.7 log_10_ between concentrations (0–80 mM, 5.26 ± 0.10–5.60 ± 0.07 log_10_, *p* > 0.05). After 6 h, for SB addition, the range of *Salmonella* counts was significantly lower compared to no addition of SB (5–80 mM, *p* < 0.05), 6.78 ± 0.84–7.90 ± 0.10 log_10_ for 5 mM, and 7.53 ± 0.04–8.71 ± 0.22 log_10_ for 0 mM. Moreover, for SB concentrations of 40 and 80 mM, no difference in the range of *Salmonella* counts over 6 h was obtained (5.23 ± 0.11–5.38 ± 0.05 log_10_, *p* > 0.05), and minor *Salmonella* growth was recorded at the earliest after 24 h incubation. Growth rates for varying SB concentrations and incubation times were confirmed in a similar manner for the three serovars. Obtained results suggest that increasing SB concentrations suppress *Salmonella* growth for concentrations of 5–20 mM over a 6 h incubation period and for 40 and 80 mM over a 24 h incubation period. When transferring these *in vitro* findings to the porcine organism, it may be assumed that *Salmonella* reduction can be achieved by increased butyrate content in the chyme of the large intestine.

## Introduction

Salmonellosis, as a zoonotic disease, is considered the second most commonly occurring bacterial gastrointestinal disease in humans worldwide (1–3). Additionally, antimicrobial resistance is emerging for *Salmonella*, which emphasizes the need to control it differently (4–6). The pathogen is able to pass through the entire food chain from stable to table: beginning at the stage of livestock farming, representing primary production, and moving on to food processing facilities and households (7, 8). Among livestock, pigs are one of the main sources of *Salmonella* infection for humans (9–11). At the farm level, they can cause an infection in people in close contact (12–14). Furthermore, in the food chain, human Salmonellosis can develop through pork meat consumption (7, 15, 16), which is described as the most frequent route of infection (11, 17). For this reason, the EU Zoonoses Regulation (EC. No. 2160/2003) targets *Salmonella* reduction with emphasis on primary production, reducing the number of *Salmonella* cases in the feed-to-food chain (2). Whereas humans are affected with clinical symptoms (3, 18, 19), pigs are mainly diagnosed asymptomatically with intermittent shedding (20), making it even harder to control the zoonotic pathogen. Results of various studies showed that an improved hygiene concept does not necessarily result in a decrease in *Salmonella* prevalence (21, 22). Due to this controversial state of facts, great value is placed on control strategies beyond hygiene measures. Even with decreasing or consistent numbers (3), foodborne *Salmonella* infections still occur, which shows there is a demand for implementing and testing a combination of reduction strategies for the zoonotic pathogen as the ability to act is increasingly limited.

Short- and medium-chain fatty acids have moved into the focus of research to reduce *Salmonella* in livestock animals (23–26). The short-chain fatty acid (SCFA) butyric acid, which is present in the porcine hindgut lumen in the dissociated form as butyrate, is attributed to exert numerous positive effects on the gastrointestinal health of livestock animals (27, 28). First, butyrate is used to supply the enterocytes with energy (29, 30), enhances the epithelial barrier function (31, 32), and downregulates the inflammation rate of the intestine (33, 34). This, in turn, leads to increased intestinal health, making it more difficult for pathogens to colonize. In the specific case of *Salmonella*, a decrease in invasion genes on *Salmonella* Pathogenicity Island 1 (SPI-1) was identified for butyrate (35–38), challenging the pathogen to penetrate the epithelial cell. Therefore, it may be assumed that bacterium resides even longer in the intestinal contents and is exposed to butyrate, which, on the one hand, is continuously infused with ingesta or generated by fermenting microorganisms (27, 28). Substituting butyric acid, sodium butyrate (SB) is already used as a feed additive in pig nutrition due to its antimicrobial properties on *Salmonella* (38–42). SCFA concentrations are greatest in the cecum and colon, which are the main fermentation sites in mammals (43). According to *in vivo* studies, the concentration of butyrate in the colon chyme of pigs differs from 9.5 up to 23.9 mM/kg (44–47). Studies carried out on fistulated pigs have shown that the majority of infused SCFA are not excreted by the animal *via* feces (48). A fast metabolism of the SCFA by intestinal microbiota and absorption by the colonic epithelium is underlined by lower butyrate concentrations found in portal venous blood (27, 28).

To investigate differences in the effectiveness of the applied control strategy because of diverse pathogenicities and growth kinetics of varying *Salmonella* serovars (49–51), a panel of three *Salmonella enterica* serovars was examined. The serovars *S*. Typhimurium (4,5:i:1,2) and *S*. Derby (4:f,g) are highly prevalent in European pigs (9, 11, 52) and are also among the generalists considered to become a human health problem in the near future (15).

Earlier studies, investigating the *in vitro* effect of butyrate on non-host-specific *Salmonella*, used a single butyrate concentration between 10 and 100 mM and incubation times of 2, 4, 6, and 24 h (35–37, 53). In the present study, however, an increasing range of concentrations and incubation times following the previous studies were used to determine the SB concentration and incubation time in the porcine colonic lumen with the highest efficacy to mitigate the growth of pig-associated *Salmonella* and to evaluate differences between serovars. SB concentrations of 0, 5, 10, 20, 40, and 80 mM were used at incubation times of 0, 1, 2, 4, 6, and 24 h applied to three *Salmonella* serovars. A pH value of 6.0 was established to model the pig's hindgut conditions (46, 54–56).

## Materials and methods

The experiment was conducted in the S2 Laboratories at the University of Veterinary Medicine Foundation, Hannover, Germany.

### Bacterial strains and inoculum preparation

Three *Salmonella enterica* serovars were used in this study. Two of them, *S*. Derby (4:f,g) and *S*. Typhimurium (4,5:i:1,2), originated from a sow herd participating in a project connected to the trials and were identified and preserved by AniCon Labor GmbH (AniCon Labor GmbH, Höltinghausen, Germany). Additionally, *S*. Typhimurium DSM 19587 (*S*. Typhimurium DSM) was used as a control strain (German collection of microorganisms and cell cultures, Braunschweig, Germany). To prepare the bacterial suspension, the strains were plated out on Columbia agar with 5% sheep blood (Thermo Scientific™, Thermo Fischer Scientific GmbH, Wesel, Germany) for 24 h at 37 °C. After incubation, a bacterial suspension with a concentration of 8.0 log_10_ CFU/mL was obtained by suspending one colony in sterile sodium chloride (NaCl) solution and adjusted until a 0.5 McFarland standard was reached (McFarland densitometer DEN-1B, BioSan SIA, Riga, Latvia). Simultaneously, the bacterial concentration of the inoculated dose was verified by direct plating of appropriate dilutions of the suspension on Xylose Lysine Deoxycholate selective agar (XLD, Thermo Scientific™, Thermo Fisher Scientific GmbH).

### Experimental set up

**Figure 1:**
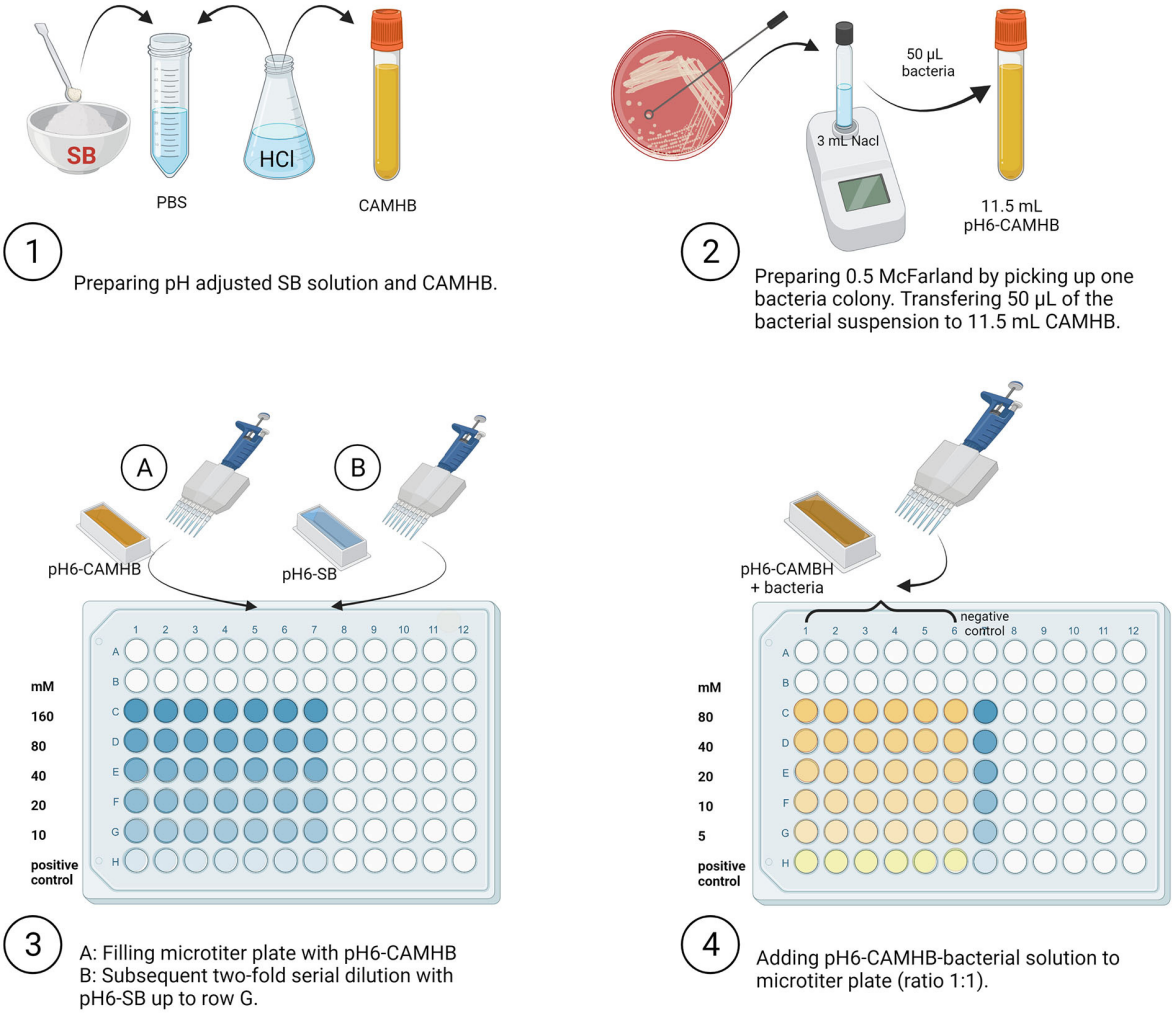
Overview of experimental set-up. SB, Sodium butyrate; PBS, Phosphate Buffer Saline; HCl, Hydrochloric acid; CAMHB, Cationic Adjusted Mueller-Hinton broth; NaCl, Sodium chloride (Created with BioRender.com).

The effect of different concentrations of SB (Carl Roth GmbH & Co. KG, Karlsruhe, Germany) on the three *Salmonella* strains at a constant pH value of 6.0 was determined using broth microdilution assay following the Clinical and Laboratory Standards Institute's recommendation for inoculum density, growth medium, and incubation times (57); the experimental set-up is shown in Figure 1.

Briefly, SB was prepared in Phosphate Buffer Saline (PBS, Thermo Fisher Scientific GmbH) by adding 0.73 g/2.5 mL to obtain 2,560 mM SB. Cationic Adjusted Mueller-Hinton broth (CAMBH, MERLIN Diagnostika GmbH, Berlin, Germany) was used as the culture medium for *Salmonella*. The pH value of all solutions and broth culture media was adjusted to the pH value of 6.0 by adding hydrochloric acid (HCl). The pH value was subsequently determined using a calibrated glass electrode (HI 2211 pH/ORP Meter, Hanna Instruments Deutschland GmbH, Vöhringen, Germany). The amount of HCl to be added was estimated in a preliminary trial (SB concentration after pH adjustment was 1,280 mM). The SB dilution step was performed in a 96-well microtiter plate (Sarstedt AG & Co, Nuembrecht, Germany), previously filled with 50 μL pH-adjusted CAMBH for each well. Fifty microliters of formerly prepared pH-adjusted SB was added to the first row (A) of the microtiter plate and then further transferred to obtain 2-fold dilutions of 160, 80, 40, 20, and 10 mM. Dilutions were prepared at twice the required final concentration because the addition of equal CAMHB-inoculum-volume reduced the concentration in half. The last row (H) contained no SB and was used as the positive control for *Salmonella* growth. Subsequently, for each strain, a volume of 50 μL from the prepared NaCl suspension containing the *Salmonella* strain (~8.0 log_10_ CFU/mL) was added to 11.5 mL pH adjusted CAMHB to obtain ~5.7 log_10_ *Salmonella* CFU/mL. From this suspension, 50 μL was distributed in each well except for the negative controls (only SB 2-fold dilutions). At this step, SB concentrations were 80, 40, 20, 10, 5, and 0 mM.

To cover six defined time points/durations of incubation (0, 1, 2, 4, 6, and 24 h), the above-described procedure was performed six times on the microtiter plate. Subsequently, the plate was incubated at 37 °C for the specified durations under aerobic conditions. The experiment was repeated three times with all serovars, concentrations, and incubation (*n* = 3).

### *Salmonella* detection method

**Figure 2:**
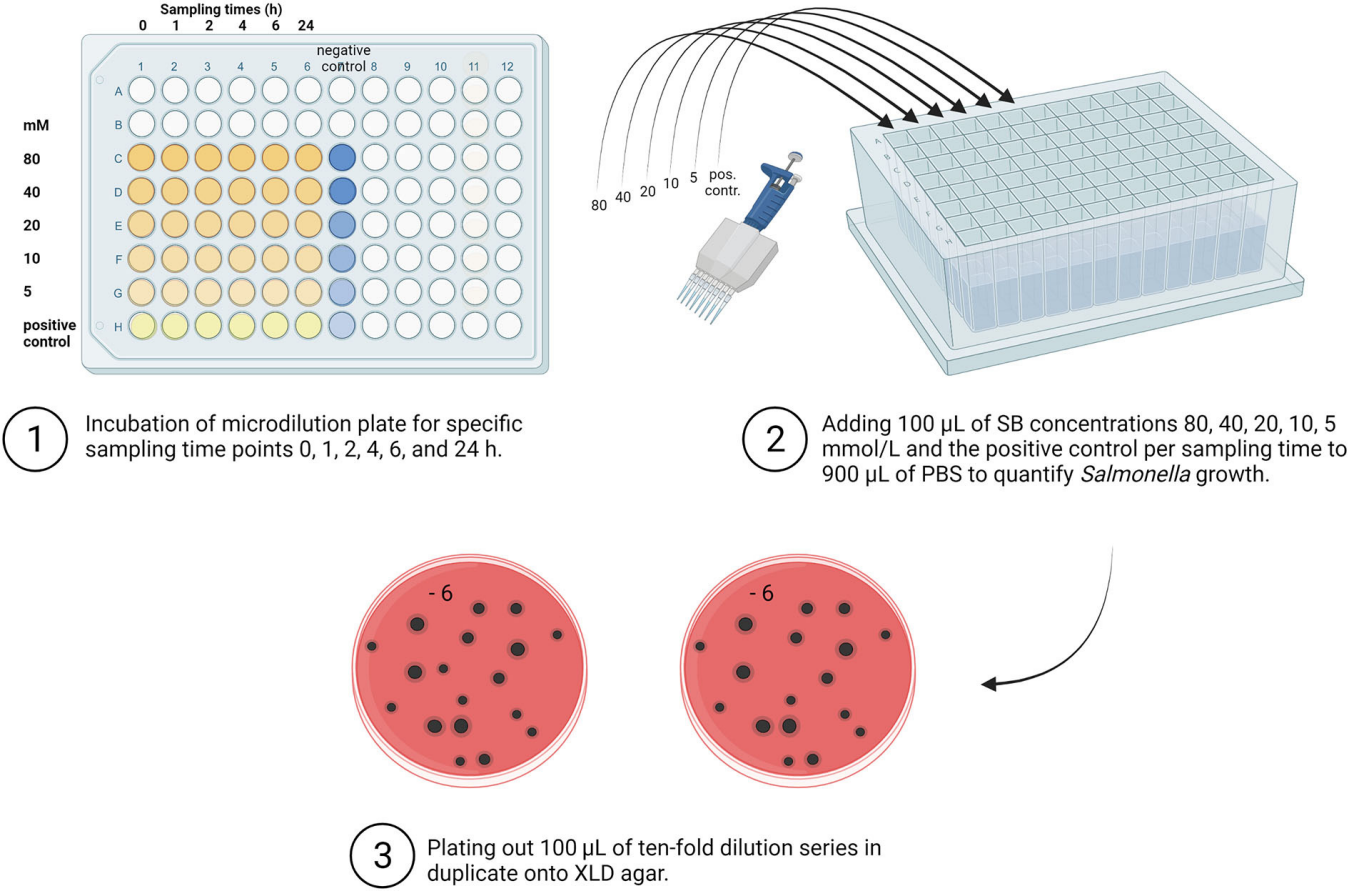
Overview of quantitative *Salmonella* detection. SB, Sodium butyrate; PBS, Phosphate Buffer Saline; XLD, Xylose Lysine Deoxycholate (Created with BioRender.com).

The *Salmonella* investigations were carried out for all samples quantitatively in accordance with DIN EN ISO 6579 (58). The following steps are shown in Figure 2.

As a first dilution step at each sampling time for each examined concentration (80, 40, 20, 10, 5, and 0 mM), 100 μL from the incubated microtiter plates were used for the quantitative determination of *Salmonella*. The incubated solution was transferred to a deep-well block (96 well MegaBlock, RTM, 2.2 ml, Sarstedt AG & Co, Nümbrecht) previously filled with 900 μL PBS (Thermo Fisher Scientific GmbH, Germany). Quantitative detection of *Salmonella* was carried out using the plate counting method. For further dilution, the previously performed first dilution step in the deep-well block was again 10-fold serially diluted to gain appropriately diluted sample suspensions. In the following step, aliquots (100 μL) of appropriate dilutions were plated in duplicate onto an XLD agar (Thermo Fisher Scientific GmbH). After incubating the media for 24 h at 37 °C, the characteristic black *Salmonella* colonies were counted, and the results were expressed in log_10_ CFU/mL.

### Chemical analysis

The butyrate content was measured for each of the incubated *Salmonella*-SB-solutions by gas chromatography using GC Shimadzu FID 2014 (Shimadzu, Kyoto, Japan) with a Stabilwax-DA capillary column, 30 m, 0.32 mm ID, 0.50 μm (Restek Corporation, Bellefonte, PE, USA). The samples were mixed 1:10 with an internal standard (17% phosphoric acid with 4-methylvaleric acid) and subsequently stored at −80 °C. After defrosting, samples were centrifuged for 15 min at 3,000 rpm, diluted at 1:4 with H_2_O, and afterward analyzed. Samples were subjected to gas chromatography with an oven temperature of 80 °C (held for 1 min) and heated to 225 °C at 8.5 °C/min (held for 5 min). N_2_ was used as a carrier.

### Statistical evaluation

For statistical evaluation, the SAS software package version 7.1 (SAS Institute, Cary, NC, USA) was used, supported by the Institute for Biometry, Epidemiology and Information Processing, University of Veterinary Medicine Hannover Foundation. The growth rate, which served as a parameter to compare the three serovars, was calculated as the difference of the bacterial counts of the five measuring points (1, 2, 4, 6, and 24 h) to the initial bacterial counts (0 h). Measurements such as mean values and standard deviations were calculated for descriptive statistics. Assuming that all values were normally distributed, data were tested for significant differences of log_10_ CFU/mL for each *Salmonella* strain using a one-way analysis of variance (ANOVA) with multiple comparisons according to Ryan-Einot-Gabriel-Welsch. To identify the influence of the incubation time, a mixed ANOVA with time as a fixed effect was used. Differences with a significant level of *p* < 0.05 were considered significant.

## Results

### *Salmonella* growth depending on sodium butyrate concentration and incubation time

**Figure 3:**
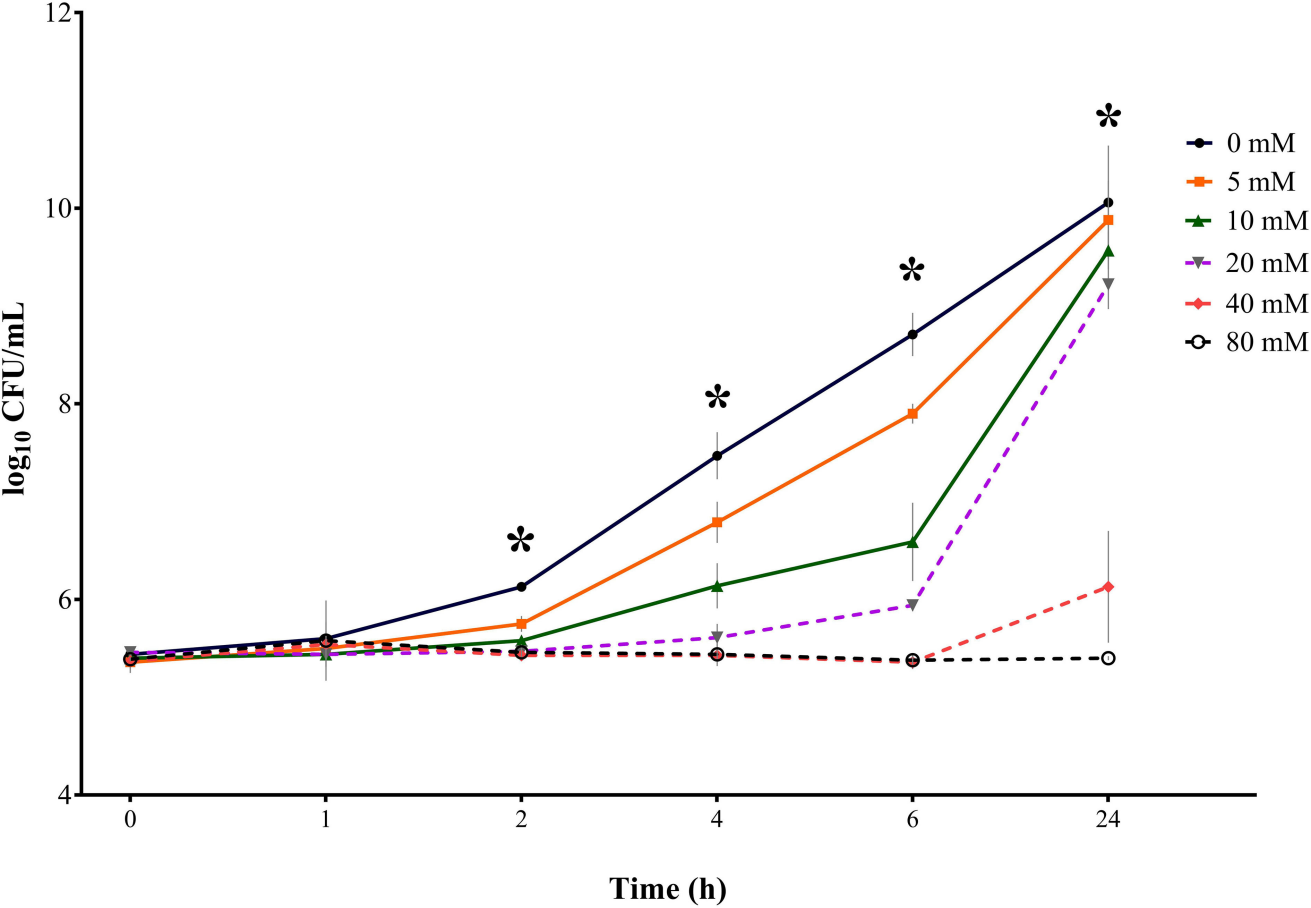
Bacterial growth curves (log_10_ CFU/mL) for *S*. Typhimurium DSM 19587 for three consecutive trials (*n* = 3) for sodium butyrate (SB) concentrations 0, 5, 10, 20, 40, and 80 mM and incubation times 0, 1, 2, 4, 6, and 24 h; * indicates significant difference between concentrations (*p* < 0.05).

**Figure 4:**
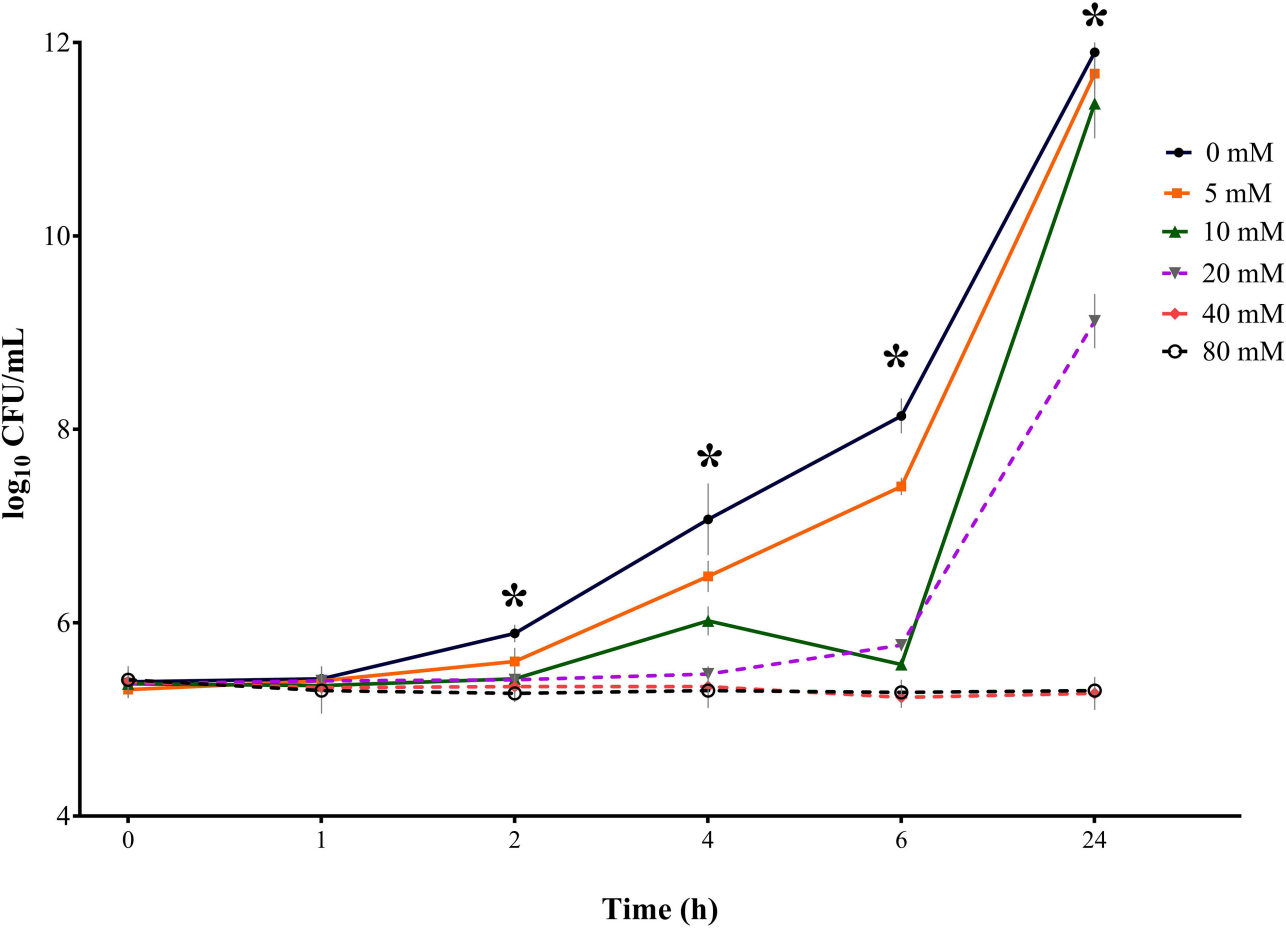
Bacterial growth curves (log_10_ CFU/mL) for *S*. Derby for three consecutive trials (*n* = 3) for sodium butyrate (SB) concentrations 0, 5, 10, 20, 40, and 80 mM and incubation times 0, 1, 2, 4, 6, and 24 h; * indicates significant difference between concentrations (*p* < 0.05).

**Figure 5:**
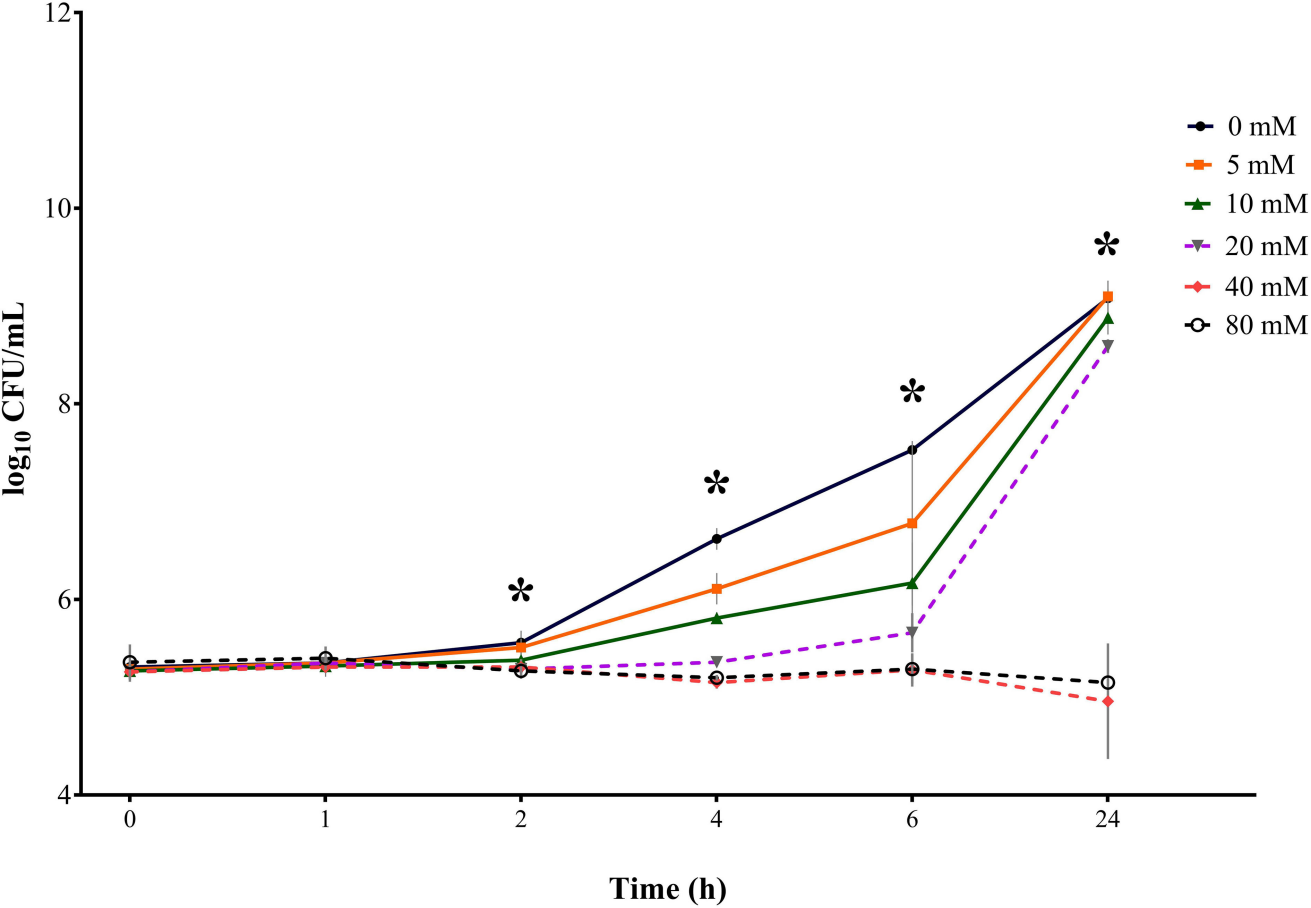
Bacterial growth curves (log_10_ CFU/mL) for *S*. Typhimurium for three consecutive trials (*n* = 3) for sodium butyrate (SB) concentrations 0, 5, 10, 20, 40, and 80 mM and incubation times 0, 1, 2, 4, 6, and 24 h; *indicates significant difference between concentrations (*p* < 0.05).

**Figure 6:**
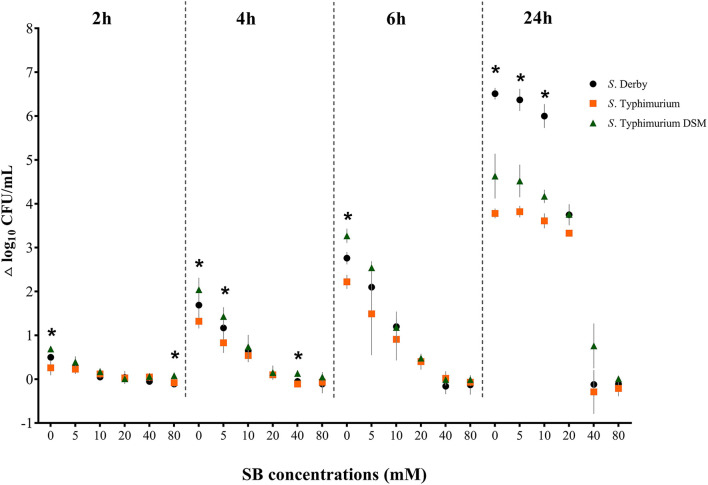
Growth rate (Δ log_10_ CFU/mL) for the serovars *S*. Derby (circle), *S*. Typhimurium (square) and *S*. Typhimurium DSM 19587 (triangle) for three consecutive trials (*n* = 3) for sodium butyrate (SB) concentrations 0, 5, 10, 20, 40, and 80 mM divided by incubation duration 2, 4, 6 and 24 h; *indicates significant difference between serovars (*p* < 0.05).

Results of *Salmonella* growth divided by serovar regarding SB concentrations and incubation time are summarized in Tables 1–**3** and Figures 3–**5**. The comparison of the three serovars is shown in **Figure 6** and Supplementary Table S1. For all three serovars, the concentration of 0 mM served as a positive control. The bacterial counts for different SB concentrations increasing in duplicate were compared for each strain.

**Table 1:** Bacterial counts (mean ± SD) of *S*. Typhimurium DSM 19587 in log_10_ CFU/mL in CAMHB^1^ adjusted to a pH value of 6.0 exposed to different sodium butyrate concentrations at different incubation times, showing influence of concentration and incubation time (*n* = 3).

**Incubation time**	**Sodium butyrate concentration in mM**						***p*** **-value**
	0	5	10	20	40	80	
	***S***. **Typhimurium DSM 19587**						
0 h	5.44^aE^ ± 0.07	5.36^aE^ ± 0.11	5.40^aD^ ± 0.04	5.46^aC^ ± 0.04	5.37^aB^ ± 0.07	5.39^aA^ ± 0.02	0.4029
1 h	5.60^aE^ ± 0.07	5.50^aDE^ ± 0.09	5.44^aD^ ± 0.06	5.44^aC^ ± 0.12	5.54^aB^ ± 0.23	5.58^aA^ ± 0.40	0.8838
2 h	6.13^aD^ ± 0.05	5.75^bD^ ± 0.08	5.58^cD^ ± 0.04	5.47^cC^ ± 0.07	5.43^cB^ ± 0.03	5.46^cA^ ± 0.06	<0.0001
4 h	7.47^aC^ ± 0.24	6.79^bC^ ± 0.21	6.14^cC^ ± 0.23	5.61^dC^ ± 0.14	5.43^dB^ ± 0.04	5.44^dA^ ± 0.12	<0.0001
6 h	8.71^aB^ ± 0.22	7.90^bB^ ± 0.10	6.59^cB^ ± 0.40	5.94^dB^ ± 0.06	5.36^eB^ ± 0.07	5.38^eA^ ± 0.05	<0.0001
24 h	10.1^aA^ ± 0.58	9.88^aA^ ± 0.40	9.57^aA^ ± 0.19	9.22^aA^ ± 0.25	6.13^bA^ ± 0.57	5.40^bA^ ± 0.02	<0.0001
*p*-value	<0.0001	<0.0001	<0.0001	<0.0001	0.0375	0.7213	

^1^CAMHB: Cationic-adjusted Mueller Hinton bouillon.

^a−e^Different subscripts within a row mark significant differences between concentrations (p < 0.05).

^A−E^Different subscripts within a column mark significant differences between incubation times (p < 0.05).

#### *S*. Typhimurium DSM 19587

Results for control serovar *S*. Typhimurium DSM 19587 (Table 1, Figure 3) showed no significant difference in bacterial counts for SB concentrations for incubation times 0 and 1 h. At incubation times of 2, 4, 6, and 24 h, a significant difference in decreasing *Salmonella* counts for higher SB concentrations was shown (*p* < 0.0001). After 2 h incubation, the highest counts for the positive control were obtained (6.13 log_10_), which were significantly higher than counts for 5 mM (5.75 log_10_), which, in turn, were significantly higher than counts for 10, 20, 40, and 80 mM (5.58, 5.47, 5.43, and 5.44 log_10_, respectively). When looking at 4 h, the positive control showed the highest counts (7.47 log_10_), which was significantly higher than the counts for 5 mM (6.79 log_10_), which in turn, differed significantly from counts for 10 mM (6.14 log_10_). At this time, the significantly lowest counts were obtained for concentrations of 20, 40, and 80 mM (5.61, 5.43, and 5.44 log_10_, respectively). For 6 h incubation, the positive control showed the significantly highest growth (8.71 log_10_), followed by 5 mM (7.90 log_10_), 10 mM (6.59 log_10_), and 20 mM (5.94 log_10_), which were all significantly different from each other. Whereas concentrations of 40 and 80 mM obtained the significantly lowest counts (5.36 and 5.38 log_10_, respectively). After 24 h incubation, the positive control again showed the maximum growth (10.1 log_10_), which was not significantly different from counts of 5, 10, and 20 mM anymore (9.88, 9.57, and 9.22 log_10_, respectively), whereas counts for 40 and 80 mM were still significantly low (6.13, and 5.40 log_10_, respectively).

In terms of time the following counts of *S*. Typhimurium DSM 19587 could be obtained: for the positive control, 5, 10, and 20 mM, the incubation time had a significant effect (*p* < 0.0001) on *Salmonella* counts. Only for this serovar, the incubation time has a significant impact (*p* = 0.0375) on bacterial growth at the concentration of 40 mM. For SB concentrations of 80 mM, no significant influence of time was observed. The positive control increased within 24 h from 5.44 to 10.08 log_10_. Values for 0 and 1 h did not show a significant difference but began to increase significantly at 2 h for each subsequent measuring point. For a concentration of 5 mM, a similar time effect was observed for the positive control: counts started to differ significantly at 2 h incubation and increased significantly every subsequent measuring point up to 9.88 log_10_ at 24 h. The concentration of 10 mM started to obtain significantly higher counts at 4 h, which, in turn, were significantly higher than the following measuring points. When looking at values of 20 mM, bacterial counts only started to differ significantly at 6 h and increased at 24 h. Results for 40 mM remained close to counts obtained at 0 h and only differed significantly after 24 h when counts increased significantly up to 6.13 log_10_. The results for 40 and 80 mM showed no difference in counts at the 6 h incubation period but counts remained similar after 24 h (5.38 log_10_ and 5.40 log_10_, respectively).

#### *S*. Derby

Results for *S*. Derby (Table 2, Figure 4) indicated no significant difference in bacterial growth when comparing the SB concentrations with the positive control for incubation times 0 and 1 h. After 2 h of incubation, a significant difference in bacterial counts (*p* = 0.0003) was detected between concentrations. For 4, 6, and 24 h a significant difference (*p* < 0.0001) in *Salmonella* growth between SB concentrations was confirmed. The higher the SB concentration, the lower the counts for *Salmonella*. When having a closer look at the incubation time of 2 h, all of the SB concentrations used differed significantly from the positive control regarding the highest counts (5.89 log_10_), whereas the lowest counts were seen for the SB concentration of 80 mM (5.27 log_10_). Counts for 5, 10, 20, and 40 mM already started differing significantly from one another (5.60, 5.42, 5.41, and 5.34 log_10_, respectively). At an incubation time of 4 h, the positive control again showed the maximum counts (7.07 log_10_) and significantly lower counts recorded for increasing concentrations of 5 to 80 mM (6.48, 6.02, 5.47, 5.34, and 5.30 log_10_, respectively). Similarly, for an incubation time of 6 h, the obtained counts were significantly lower in ascending order of concentration. Again, the positive control had the maximum *Salmonella* counts (8.14 log_10_), with significantly higher counts compared to 5 mM (7.41 log_10_) and even significantly higher counts compared to 10 mM (6.57 log_10_) and, in turn, significantly higher counts compared to 20 mM (5.77 log_10_). The significant lowest counts were obtained at 40 and 80 mM (5.23 and 5.28 log_10_, respectively). After 24 h of incubation, the maximum growth was still detected for the positive control (11.90 log_10_); however, it was not significantly different anymore from counts of 5 and 10 mM (11.68 and 11.37 log_10_, respectively). The *Salmonella* counts of the positive control, 5, and 10 mM were followed by significantly lower counts for 20 mM (9.12 log_10_), which, in turn, differed significantly from *Salmonella* counts, with the minimum numbers being expressed for concentrations 40 and 80 mM (5.27 and 5.30 log_10_, respectively).

**Table 2:** Bacterial counts (mean ± SD) of *S*. Derby in log_10_ CFU/mL in CAMHB^1^ adjusted to a pH value of 6.0 exposed to different sodium butyrate concentrations at different incubation times, showing influence of concentration and incubation time (*n* = 3).

**Incubation time**	**Sodium butyrate concentration in mM**						***p*** **-value**
	0	5	10	20	40	80	
	***S***. **Derby**						
0 h	5.39^aE^ ± 0.16	5.31^aE^ ± 0.09	5.37^aD^ ± 0.11	5.37^aC^ ± 0.07	5.39^aA^ ± 0.13	5.41^aA^ ± 0.09	0.9152
1 h	5.42^aE^ ± 0.13	5.40^aE^ ± 0.13	5.35^aD^ ± 0.11	5.40^aC^ ± 0.10	5.33^aA^ ± 0.12	5.30^aA^ ± 0.24	0.9059
2 h	5.89^aD^ ± 0.09	5.60^bD^ ± 0.14	5.42^bcD^ ± 0.11	5.41^bcC^ ± 0.13	5.34^bcA^ ± 0.12	5.27^cA^ ± 0.09	0.0003
4 h	7.07^aC^ ± 0.37	6.48^bC^ ± 0.16	6.02^cC^ ± 0.15	5.47^dC^ ± 0.08	5.34^dA^ ± 0.06	5.30^dA^ ± 0.18	<0.0001
6 h	8.14^aB^ ± 0.18	7.41^bB^ ± 0.09	6.57^cB^ ± 0.05	5.77^dB^ ± 0.06	5.23^eA^ ± 0.11	5.28^eA^ ± 0.13	<0.0001
24 h	11.90^aA^ ± 0.16	11.68^aA^ ± 0.16	11.37^aA^ ± 0.36	9.12^bA^ ± 0.28	5.27^cA^ ± 0.17	5.30^cA^ ± 0.06	<0.0001
*p*-value	<0.0001	<0.0001	<0.0001	<0.0001	0.6659	0.7987	

^1^CAMHB: Cationic-adjusted Mueller Hinton bouillon.

^a−e^Different subscripts within a row mark significant differences between concentrations (p < 0.05).

^A−E^Different subscripts within a column mark significant differences between incubation times (p < 0.05).

When looking at results for *S*. Derby regarding the influence of incubation time within one concentration, a significant effect (*p* < 0.0001) of incubation time was found on *Salmonella* counts during the measured period for the positive control, 5, 10, and 20 mM but no significant time effect was apparent for 40 and 80 mM (*p* > 0.05). Within 24 h, the counts for the positive control increased from 5.39 log_10_ to 11.90 log_10_. Except for 0 and 1 h, counts for the positive control showed significantly higher counts at each subsequent measuring point. For 5 mM, similar findings were obtained. The SB concentration of 10 mM did not show a significant increase in counts for 2 h incubation but started to rise significantly from measuring point 4 h onwards. When considering the values for 20 mM, a significant increase in *Salmonella* counts was only seen from 6 h onwards, with up to counts of 9.12 log_10_ at 24 h. Looking at the highest SB concentrations 40 and 80 mM, even a slight decrease in bacterial counts, which was not significant, was found for the observed period. For both concentrations within the 24 h period, no significance was found in *Salmonella* counts at any point.

#### *S*. Typhimurium

Results for *S*. Typhimurium ((Table 3, Figure 5) were similar to results for the control-serovar and *S*. Derby. No significant difference in bacterial counts for SB concentrations was detected for incubation times 0 and 1 h. Growth started to differ significantly from 2 h onwards (*p* = 0.0040). At incubation times of 4, 6, and 24 h, the significance of decreasing *Salmonella* counts for higher SB concentrations was determined (*p*<0.0001). After incubation of 2 h, maximum counts were recovered from the positive control (5.56 log_10_), significantly lower counts for 5 and 10 mM (5.51 and 5.38 log_10_. respectively), whereas, in turn, significantly lower counts at a minimum stage were observed for 40 and 80 mM (5.31 and 5.27 log_10_, respectively). After 4 h incubation, the highest significant counts for *S*. Typhimurium growth were again observed for the positive control (6.62 log_10_). At this time, the positive control differed significantly from counts of 5 mM (6.11 log_10_), which differed significantly from counts of 10 mM (5.81 log_10_), which, in turn, differed significantly from minimum counts of 20, 40, and 80 mM (5.36, 5.15, and 5.20 log_10_, respectively). At incubation time of 6 h, counts for the positive control increased continuously to maximum numbers (7.53 log_10_), which were significantly higher compared to counts for 5 and 10 mM (6.78 and 6.17 log_10_, respectively), whereas counts for 20, 40, and 80 mM remained significantly lower at a minimum level (5.66, 5.28, and 5.29 log_10_, respectively). After 24 h, the highest counts were detected for 5 mM (9.10 log_10_), whereas these were not significantly different from the positive control, 10, and 20 mM (9.08, 8.88, and 8.59 log_10_, respectively). Meanwhile, these were significantly higher compared to the lowest counts of 40 and 80 mM (4.96 and 5.15 log_10_, respectively).

**Table 3:** Bacterial counts (mean ± SD) of *S*. Typhimurium in log_10_ CFU/mL in CAMHB^1^ adjusted to a pH value of 6.0 exposed to different sodium butyrate concentrations at different incubation times, showing influence of concentration and incubation time (*n* = 3).

**Incubation time**	**Sodium butyrate concentration in mM**						***p*** **-value**
	0	5	10	20	40	80	
	***S***. **Typhimurium**						
0 h	5.31^aE^ ± 0.13	5.29^aD^ ± 0.10	5.27^aD^ ± 0.09	5.26^aC^ ± 0.08	5.26^aA^ ± 0.10	5.36^aA^ ± 0.18	0.8960
1 h	5.35^aE^ ± 0.10	5.35^aD^ ± 0.14	5.32^aD^ ± 0.07	5.35^aC^ ± 0.06	5.31^aA^ ± 0.06	5.40^aA^ ± 0.12	0.8824
2 h	5.56^aD^ ± 0.12	5.51^abCD^ ± 0.09	5.38^abcD^ ± 0.01	5.29^cC^ ± 0.10	5.31^bcA^ ± 0.05	5.27^cAB^ ± 0.08	0.0040
4 h	6.62^aC^ ± 0.11	6.11^bBC^ ± 0.16	5.81^cC^ ± 0.06	5.36^dC^ ± 0.03	5.15^dA^ ± 0.06	5.20^dAB^ ± 0.03	<0.0001
6 h	7.53^aB^ ± 0.04	6.78^abB^ ± 0.84	6.17^bcB^ ± 0.41	5.66^cB^ ± 0.20	5.28^cA^ ± 0.17	5.29^cAB^ ± 0.15	<0.0001
24 h	9.08^aA^ ± 0.18	9.10^aA^ ± 0.07	8.88^aA^ ± 0.17	8.59^aA^ ± 0.07	4.96^bA^ ± 0.59	5.15^bB^ ± 0.03	<0.0001
*p*-value	<0.0001	<0.0001	<0.0001	<0.0001	0.4171	0.1318	

^1^CAMHB: Cationic-adjusted Mueller Hinton bouillon.

^a−e^Different subscripts within a row mark significant differences between concentrations (p < 0.05).

^A−E^Different subscripts within a column mark significant differences between incubation times (p < 0.05).

When looking at values for *S*. Typhimurium in terms of incubation time, similar findings compared to *S*. Typhimurium DSM 19587 and *S*. Derby could be observed. For the positive control, 5, 10, and 20 mM the incubation time had a significant effect (*p* < 0.0001) on *Salmonella* counts. For SB concentrations of 40 and 80 mM, no significant influence of time was observed. The positive control increased after 24 h incubation from 5.31 to 9.08 log_10_. Values for 0 and 1 h did not show a significant difference but began to increase significantly at 2 h incubation and for each subsequent measuring point. The following concentrations of 5 mM and 10 mM started to differ in counts significantly as well at 2 h rising subsequently up to 24 h. When looking at 20 mM, counts only started to increase significantly from 6 h, with the highest counts being reached at 24 h. The concentration of 40 mM showed a decrease in *Salmonella* counts when comparing 0 h with 24 h; however, time had no significant influence (*p* = 0.4171). Whereas 80 mM showed a significant decrease when comparing counts at 2, 4, 6, and 24 h, this did not result due to a general significant effect (*p* = 0.1318) of incubation time for this concentration.

### Comparison of the three serovars

Figure 6 shows the *Salmonella* growth rate subdivided according to incubation duration, comparing the three serovars *S*. Typhimurium DSM 19587, *S*. Derby and *S*. Typhimurium. Complete data can be found in Supplementary Table S1. The growth rates described here refer to the time points 2, 4, 6, and 24 h, at which a significant difference in counts was observed. Overall, when considering the incubation periods and serovars, the bacterial growth rate was negatively affected by increasing SB concentrations. Without the addition of SB, there was a significant difference in the range of growth rates during the investigated 24 h between the serovars, which became progressively greater as time proceeded (Δ_2−24*h*/0*mM*_: *S*. Typhimurium DSM 0.69–4.63 log_10_, *S*. Derby 0.50–6.51 log_10_, *S*. Typhimurium 0.26–3.78 log_10_; *p* < 0.05). After 2 h incubation, a significant difference in growth rates between the serovars was also seen for 80 mM (Δ_2h/80mM_: *S*. Typhimurium DSM 0.08 log_10_, *S*. Typhimurium −0.08 log_10_, *S*. Derby −0.14 log_10_; *p* = 0.0237). Incubating the serovars for 4 h showed a significant difference in growth rates between the serovars as well for 5 mM (Δ_4h/5mM_: *S*. Typhimurium DSM 1.43 log_10_, *S*. Derby 1.17 log_10_, *S*. Typhimurium 0.83 log_10_; *p* = 0.0264) and 40 mM (Δ_4h/40mM_: *S*. Typhimurium DSM 0.13 log_10_, *S*. Derby −0.05 log_10_, *S*. Typhimurium −0.11 log_10_; *p* = 0.0119). After 6 h incubation, the quantitative growth rate only differed significantly between the serovars for the positive control. Whereas after 24 h incubation, the range of growth rates for 5, 10, and 20 mM increased for all serovars and differed not only significantly between the serovars for the positive control but also for 5 and 10 mM (Δ_24h/0−10mM_: *S*. Derby 6.51–6.00 log_10_, *S*. Typhimurium DSM 4.63–4.17 log_10_, *S*. Typhimurium 3.82–3.61 log_10_; *p* < 0.0001). For concentrations of 40 and 80 mM, in some cases, even a negative growth rate was obtained.

### Chemical analysis

The results of the butyrate analysis after each incubation period for three consecutive trials were measured. Briefly, even with a prolonged incubation period, no change in the butyrate concentration could be detected. Analyzed values were higher than the calculated concentrations. However, the analyzed butyrate values halved as did the calculated values. Only results for *S*. Typhimurium DSM are shown in Supplementary Table S2, as the results for *S*. Derby and *S*. Typhimurium were not different. The determination of the pH value confirmed values of 6.0 for all solutions (data not shown).

## Discussion

The *in vitro* study showed that sodium butyrate (SB) has a mitigating effect on *Salmonella*, which is even more effective with the increasing amount of SB. The comparison of the serovars led to the assumption that this effect applies equally to different serovars.

The pathogen, which survived the passage through the stomach and intestine, is initially located in the lumen of the colon, more precisely in the chyme, before invading the epithelial cell (15). In the intestinal content, it is exposed to butyrate, which is the situation we simulated here. Results of the *in vitro* study indicated that the addition of SB already led to significantly less bacterial growth after 2 h incubation time compared to no addition of SB. After 6 h incubation, for SB concentrations of 5–20 mM, bacterial growth was significantly decreased, whereas concentrations of 40 and 80 mM obtained even consistent counts over 6 h and showed only modest growth after 24 h incubation. The lower concentrations after 24 h showed significant *Salmonella* growth, with counts no longer different from the positive control. All the previous studies concluded that butyrate decreased the ability of *Salmonella* to invade the epithelial cell. Gantois et al. (37) found that butyrate at a concentration of 10 mM showed the least invasion of *Salmonella* after 4 h. They attributed this to the downregulation of invasion genes located on *Salmonella* Pathogenicity Island 1 (SPI-1), which is described as the *Salmonella* chromosome encoding the ability for invasion (59). Chu et al. (60) made similar observations for butyrate concentrations of 10 mM: a significantly limited intracellular proliferation of *Salmonella* after 6–12 h of incubation was obtained. Furthermore, Van Immerseel et al. (36) observed significantly reduced *Salmonella* invasion after incubating bacteria supplemented with 20 and 40 mM butyrate for 4 h. Additionally, Lawhon et al. (35) investigated the effect of butyrate on *Salmonella* and found that butyrate besides propionate at concentrations of 30 mM and pH 6.7 exerted an inhibitory effect on *Salmonella* invasion genes, whereas the most abundant SCFA acetate exerted a promoting effect. However, using a mixture of the three SCFA, containing the range of colonic SCFA but with lower ileal concentrations (a total SCFA of 30 mM, consisting of 16.5 mM acetate, 10.5 mM propionate, and 3 mM butyrate), results showed no significant effect on invasion processes. These findings were comparable to those of Van Immersel et al. (36) who added an avian *in vivo*-like cecal SCFA mixture low in terms of butyrate concentration (consisting of 33 mM acetate, 12 mM propionate, and 6 mM butyrate), which also resulted in no reduced *Salmonella* invasion. When Lawhon et al. (35) applied a colonic SCFA mixture with higher concentrations of 200 mM, the total SCFA consisting of 110 mM acetate, 70 mM propionate, and 20 mM butyrate, results indicated that the amount of propionate and butyrate had a decisive influence on the inhibition of invasion genes and consequently on *Salmonella* proliferation, which was also considered likely by Gantois et al. (37). It has to be emphasized that the above-listed literature has investigated the effect of butyrate, focusing on the invasive ability of *Salmonella*. In contrast, we concluded that the sole presence of SB reduced the growth of *Salmonella*. Nevertheless, their results in terms of concentration and incubation time are in accordance with the findings of the present *in vitro* study, which indicates the higher the SB concentrations, the lower the observed bacterial growth. Even though reducing effects at low concentrations, such as 5 and 10 mM, were obtained, it has to be mentioned that no other physiological processes potentially consuming butyrate were taken into consideration in the *in vitro* study. Therefore SB concentrations of 20 mM and higher appear to be more realistic to exert a similar mitigating effect *in vivo*. Fortunately, there is more *in situ* data on the luminal SCFA content for pigs than for humans (27). To mention only a few studies, Hedemann and Bach Knudsen (44) found colonic butyrate values of 9.5 mM/kg, while Bunte (45) and Bullermann (46) found values up to 10.8 mM/kg and 16.2 mM/kg, respectively; Wilke (47) found even higher values of 23.9 mM/kg in pigs' colon chyme for conventional feeding. On the one hand, the concentrations investigated in the present study with values of 5–80 mM were chosen on the basis of the above-listed studies, but, on the other hand, the needed butyrate content of 20 mM in the colon chyme determined in this *in vitro* study to achieve a reduction in *Salmonella* growth is not unrealistic.

The model used merely reflects the conditions in the colonic lumen. Neither host–pathogen interactions nor interactions between pathogen and the host's microbial composition were taken into account. Therefore, it is possible to only conclude a direct influence of SB on *Salmonella* without considering any side effects that may occur. It is known that an acidic pH value reduces the growth of *Salmonella* (21, 36, 61, 62). Since the pH value was constantly at 6.0 in all solutions, it can be assumed to have not influenced the growth rates discussed here.

To transfer the application of butyrate from the *in vitro* experiment further to *in vivo*, it is necessary to consider butyrate's mode of action on the organism as well. Apart from the direct effect of butyrate on *Salmonella*, it indirectly strengthens the epithelial cell, making it more difficult for a pathogen to invade (27–29), therefore speaking in favor of SB. Yan and Ayuwon (32) modeled the intestinal system using porcine epithelial cells treated with butyrate while challenging them with LPS, representing the induction of inflammation. Their results provided the initial evidence that even small quantities of 1 mM butyrate can protect cells from lipopolysaccharide (LPS)-induced increase in paracellular permeability and damage to the integrity of the epithelial barrier. A study by Nielsen et al. (31) was able to show that increased butyrate production by enrichment of butyrogenic bacteria leads to the improved barrier function of the human colonic epithelium. To conclude, following the growth-reducing results of the present *in vitro* study, the *Salmonella* invasion-reducing effects described in the literature, as well as the proven enhancement of the intestinal barrier, an increased butyrate content in the porcine hindgut should be targeted.

An increase in the SCFA content in the porcine large intestine, in particular butyrate, may, on the one hand, be achieved directly by adding SB to the feed. Especially, if the feed additive is processed by coating to protect it against its early dissociation in the stomach, the influx in the large intestine is likely to be increased. Boyen et al. (38) compared uncoated and coated SB as a feed additive. Their results showed that fecal shedding of *Salmonella* was lower for the group supplemented with the coated feed additive at a dose of 2 kg/ton feed. Casanova-Higes et al. (41) showed that feeding SB coated with the sodium salt of coconut fatty acid distillate for 3.5 months in the fattening unit at a dose of 3 kg/ton feed resulted in significantly lower *Salmonella* antibody optical density (OD)-values of serum samples and a lower proportion of positive mesenteric lymph node (MLN) samples at slaughter for treatment groups but not in fecal samples. In contrast, Walia et al. (39), who fed coated SB at the same dose of 3 kg/ton to finishing pigs 24–28 days before slaughter, did not find differences in MLN samples between treatment groups but obtained significantly lower positive fecal samples and was able to confirm lower OD-values for one of two treatment groups. However, in these three previous studies, additional measurements for the subsequent SCFA content in the pig's chyme of the large intestine are missing. According to the findings of Mallo et al. (63), the addition of 2 kg/ton coated SB led to butyrate concentrations of 8.24 mM in the colon of pigs. The dose of 3 kg/ton can be expected to lead to even higher levels, which might come closer to the levels that we have found to be effective against *Salmonella*. Besides the direct use of butyrate as a feed additive, not only Nielsen et al. (31) but also Chu et al. (60) showed that butyrate enrichment in the hindgut can be achieved by probiotics. Chu et al. added a probiotic mixture consisting of *Lactobacillus* and *Bacillus* to the feed of piglets 25 days of age and could suppress *S*. infantis from invading, which they found is due to maintaining the butyrate-producing microbiota. Furthermore, the decrease in butyrate levels was shown to be significantly reversed for probiotic addition compared to no probiotic treatment. Mild acidic pH values, as present in the colon and simulated in our study, seem to even boost the butyrate-producing bacteria, enabling them to compete with gram-negative carbohydrate utilizing bacteria such as *Bacteroides* spp. (64). Another approach to achieve higher SCFA levels may be attained by reduced grinding intensity, influencing fermentation patterns toward butyrate, as Mikkelsen et al. (65) and Visscher et al. (66) demonstrated by comparing coarsely and finely ground feed. Not only physicochemical properties seem to be crucial for microbial butyrate formation, but also using feedstuffs high in fermentable carbohydrates that specifically stimulate large intestine fermentation in the direction of butyrate production (67, 68). When Haenen et al. (69) fed pigs a diet rich in resistant starch by adding retrograded tapioca starch instead of native potato starch to the feed, subsequently, measured colonic butyrate concentrations were significantly higher. Focusing on one cereal, in particular, rye might become of great interest, as it is able to provide the microorganisms with the substrate for butyrate formation in the hindgut (70). Rye has a lower prececal digestibility, which means that a higher proportion of the original substrate reaches the large intestine (71, 72). One part of the carbohydrates, the non-starch-polysaccharides, originating from rye, largely consist of arabinoxylans and fructans, which are transformed into the three main SCFA acetate, propionate, and butyrate by microbial metabolism (73–75). A recent study by Chuppava et al. (76) has shown that feeding rye at a proportion of 69% in the compound feed to experimentally *Salmonella*-infected young pigs led to a reduction in *Salmonella* in the feces in comparison to pigs fed a diet without rye. The study by Wilke (47), who used the same compound feed, concluded that the butyrate content in the colonic chyme increased from 23.9 to 31.9 mM when 69% wheat was replaced by 69% rye.

The growth rates of the three serovars varied for the positive control over the entire incubation period. However, when SB was added, the growth rates of all serovars decreased in a similar way. This allows us to make the cautious assumption that the growth inhibitory effect also applies to *Salmonella enterica* serovars other than those used here. Furthermore, it agrees with the findings of Lianou and Koutsoumanis (49), who investigated the growth kinetics of 60 *Salmonella* strains belonging to nine serotypes. This finding is also supported by Juneja et al. (77), who observed only a negligible serovar-specific difference when investigating heat stress to 10 different *Salmonella* serovars, concluding that serotype effects are minor compared to other types of experimental variability.

Even though the results shown here were only obtained in a less complex *in vitro* model, they support the hypotheses that increasing SB concentrations reduce *Salmonella* growth, thus emphasizing the potential of SB. Our findings indicate that reaching high levels of butyrate combined with sufficient exposure times of 4–6 h studied *in vitro* exert the mitigating effect on *Salmonella* also in the large intestine *in vivo*. Further research is needed to simulate the complexity of an *in vivo Salmonella* infection with additional physiological processes of the porcine intestinal tract to substitute antibiotic treatment for dietetic measures.

## Data availability statement

The raw data supporting the conclusions of this article will be made available by the authors, without undue reservation.

## Author contributions

Conceptualization: JH, MA, and CV. Methodology: IH, JL, BC, VW, AA, JH, MA, and CV. Validation: IH, JH, MA, and CV. Formal analysis: IH and JL. Investigation and visualization: IH, MA, and BC. Data Curation: IH and MA. Writing—original draft preparation: IH. Writing—review and editing: BC, JL, AA, JB, MA, CV, IH. Supervision: MA and CV. Project administration, resources, and funding acquisition: CV. All authors have read and agreed to the published version of the manuscript. All authors contributed to the article and approved the submitted version.

## Funding

The study was supported by funds of the Federal on a decision of the Parliament of the Federal Republic of Germany via the Federal Office for Agriculture and Food (BLE, Germany) under the innovation support program, and Ministry of Food and Agriculture (BMEL, Germany). This Open Access publication was funded by the Deutsche Forschungsgemeinschaft (DFG, German Research Foundation) – 491094227 Open Access Publication Funding and the University of Veterinary Medicine Hannover, Foundation.

## Conflict of interest

Author JB is employed by Anicon Labor GmbH.

The remaining authors declare that the research was conducted in the absence of any commercial or financial relationships that could be construed as a potential conflict of interest.

## Publisher's note

All claims expressed in this article are solely those of the authors and do not necessarily represent those of their affiliated organizations, or those of the publisher, the editors and the reviewers. Any product that may be evaluated in this article, or claim that may be made by its manufacturer, is not guaranteed or endorsed by the publisher.
