# Supplementary material for: In vitro evaluation of sodium butyrate on the growth of three Salmonella serovars derived from pigs at a mild acidic pH value
Source: Front Vet Sci. 2022 Jul 26;9:937671. doi: 10.3389/fvets.2022.937671 (PMC9360501; doi:10.3389/fvets.2022.937671)
Supplement: Supplementary file 1 [file Data_Sheet_1.PDF]

Supplementary Material

Table S1. Growth rate (mean ± SD) shown as difference (Δ) of *Salmonella* counts in $\log_{10}$ CFU/mL of 2, 4, 6, and 24 h compared to counts of 0 h incubation time of three *Salmonella* serovars (*S*. Typhimurium DSM 19587, *S*. Derby, *S*. Typhimurium) in Cationic-adjusted Mueller Hinton buillon adjusted to a pH value of 6.0 exposed to different sodium butyrate (SB) concentrations (n=3)

**SB concentration in mM**	**Δ $\log_{10}$ CFU$_{2h-0h}$**				**Δ $\log_{10}$ CFU$_{4h-0h}$**				**Δ $\log_{10}$ CFU$_{6h-0h}$**				**Δ $\log_{10}$ CFU$_{24h-0h}$**			
	**serovar**				**serovar**				**serovar**				**serovar**			
	***S*. Typh.[1] DSM 19587**	***S*. Derby**	***S*. Typh.[1]**	*p*-value	***S*. Typh.[1] DSM 19587**	***S*. Derby**	***S*. Typh.[1]**	*p*-value	***S*. Typh.[1] DSM 19587**	***S*. Derby**	***S*. Typh.[1]**	*p*-value	***S*. Typh.[1] DSM 19587**	***S*. Derby**	***S*. Typh.[1]**	*p*-value
0	0.69 ^{aA} ± 0.03	0.50 ^{aA} ± 0.07	0.26 ^{bA} ± 0.17	0.0067	2.04 ^{aA} ± 0.27	1.69 ^{aA} ± 0.26	1.32 ^{bA} ± 0.16	0.0257	3.27 ^{aA} ± 0.16	2.76 ^{bA} ± 0.14	2.22 ^{cA} ± 0.16	0.0005	4.63 ^{bA} ± 0.51	6.51 ^{aA} ± 0.13	3.78 ^{cA} ± 0.11	0.0001
5	0.39 ^{aB} ± 0.13	0.29 ^{aB} ± 0.17	0.23 ^{aAB} ± 0.04	0.3453	1.43 ^{aB} ± 0.21	1.17 ^{abB} ± 0.15	0.83 ^{bB} ± 0.23	0.0264	2.54 ^{aB} ± 0.15	2.10 ^{aB} ± 0.11	1.49 ^{aAB} ± 0.94	0.1432	4.52 ^{bA} ± 0.37	6.37 ^{aAB} ± 0.25	3.82 ^{cA} ± 0.13	<0.0001
10	0.17 ^{aC} ± 0.08	0.05 ^{aC} ± 0.06	0.12 ^{aABC}± 0.10	0.2486	0.74 ^{aC} ± 0.27	0.65 ^{aC} ± 0.05	0.54 ^{aC} ± 0.15	0.4519	1.18 ^{aC} ± 0.36	1.20 ^{aC} ± 0.16	0.91 ^{aBC} ± 0.48	0.5695	4.17 ^{bAB} ± 0.15	6.00 ^{aB} ± 0.27	3.61 ^{cAB} ± 0.17	<0.0001
20	0.01 ^{aD} ± 0.09	0.04 ^{aCD}± 0.15	0.03 ^{aCD} ± 0.04	0.9501	0.15 ^{aD} ± 0.16	0.10 ^{aD} ± 0.01	0.10 ^{aD} ± 0.07	0.7529	0.48 ^{aD} ± 0.07	0.40 ^{aD} ± 0.05	0.40 ^{aCD} ± 0.18	0.6177	3.76 ^{aB} ± 0.22	3.75 ^{aC} ± 0.24	3.33 ^{bB} ± 0.04	0.0523
40	0.06 ^{aCD} ± 0.04	-0.05 ^{aCD} ± 0.08	0.05 ^{aBCD}± 0.09	0.1818	0.13 ^{aD} ± 0.08	-0.05 ^{bD} ± 0.08	-0.11 ^{bD} ± 0.04	0.0119	-0.01 ^{aE} ± 0.06	-0.16 ^{aE} ± 0.18	0.02 ^{aD} ± 0.16	0.3240	0.76 ^{aC} ± 0.51	-0.12 ^{abD} ± 0.29	-0.29 ^{bC} ± 0.50	0.0553
80	0.08 ^{aCD} ± 0.06	-0.14 ^{bD} ± 0.01	-0.08 ^{bD} ± 0.11	0.0237	0.05 ^{aD} ± 0.11	-0.11 ^{aD} ± 0.21	-0.15 ^{aD} ± 0.18	0.3851	-0.01 ^{aE} ± 0.05	-0.13 ^{aE}± 0.22	-0.07 ^{aD} ± 0.09	0.5788	0.01 ^{aD} ± 0.01	-0.11 ^{aD} ± 0.09	-0.21 ^{aC} ± 0.18	0.1400
p-value	< 0.0001	< 0.0001	< 0.0115		< 0.0001	< 0.0001	< 0.0001		< 0.0001	< 0.0001	< 0.0002		< 0.0001	< 0.0001	< 0.0001	

[1]*S*. Typh.: S. Typhimurium

$^{a, b, c}$ Different subscripts within a row mark significant differences between serovars (p< 0.05)

$^{A, B, C, D, E}$ Different subscripts within a column mark significant differences between concentrations (p< 0.05)

Table S2. Analyzed butyrate content in mM (mean ± SD) in *Salmonella*-sodium butyrate solutions incubated for 0, 1, 2, 4, 6, and 24 h for *S*. Typhimurium DSM 19587 (n=3)

Incubation time	Calculated sodium butyrate concentration in mM					
	0	5	10	20	40	80
0 h	0.08±0.06	5.39±0.64	11.14±1.30	23.53±1.19	46.53±3.59	97.60±3.82
1 h	0.11±0.07	5.53±0.61	11.33±1.19	23.07±2.32	48.00±4.20	97.33±7.75
2 h	0.14±0.11	5.71±0.69	11.40±0.78	24.23±1.96	48.33±3.18	96.70±6.11
4 h	0.11±0.04	5.96±0.79	11.70±1.32	23.60±2.76	47.73±1.21	95.47±2.26
6 h	0.07±0.02	6.94±2.50	13.47±4.03	26.57±5.27	50.47±5.25	97.60±5.67
24 h	0.11±0.07	6.15±0.99	12.63±1.80	24.40±1.93	50.00±3.36	97.43±5.33
p-value	0.7581	0.6869	0.7008	0.7291	0.7832	0.9957
